# Genetic Disruption of the *Sh3pxd2a* Gene Reveals an Essential Role in Mouse Development and the Existence of a Novel Isoform of Tks5

**DOI:** 10.1371/journal.pone.0107674

**Published:** 2014-09-26

**Authors:** Pilar Cejudo-Martin, Angela Yuen, Nicole Vlahovich, Peter Lock, Sara A. Courtneidge, Begoña Díaz

**Affiliations:** 1 Cancer Center, Tumor Microenvironment and Metastasis Program, Sanford-Burnham Medical Research Institute, La Jolla, California, United States of America; 2 La Trobe Institute for Molecular Science, La Trobe University, Victoria, Australia; Ohio State University Comprehensive Cancer Center, United States of America

## Abstract

Tks5 is a scaffold protein and Src substrate involved in cell migration and matrix degradation through its essential role in invadosome formation and function. We have previously described that Tks5 is fundamental for zebrafish neural crest cell migration *in vivo*. In the present study, we sought to investigate the function of Tks5 in mammalian development by analyzing mice mutant for *sh3pxd2a*, the gene encoding Tks5. Homozygous disruption of the *sh3pxd2a* gene by gene-trapping in mouse resulted in neonatal death and the presence of a complete cleft of the secondary palate. Interestingly, embryonic fibroblasts from homozygous gene-trap *sh3pxd2a* mice lacked only the highest molecular weight band of the characteristic Tks5 triplet observed in protein extracts, leaving the lower molecular weight bands unaffected. This finding, together with the existence of two human Expressed Sequence Tags lacking the first 5 exons of *SH3PXD2A*, made us hypothesize about the presence of a second alternative transcription start site located in intron V. We performed 5′RACE on mouse fibroblasts and isolated a new transcript of the *sh3pxd2a* gene encoding a novel Tks5 isoform, that we named Tks5β. This novel isoform diverges from the long form of Tks5 in that it lacks the PX-domain, which confers affinity for phosphatidylinositol-3,4-bisphosphate. Instead, Tks5β has a short unique amino terminal sequence encoded by the newly discovered exon 6β; this exon includes a start codon located 29 bp from the 5'-end of exon 6. Tks5β mRNA is expressed in MEFs and all mouse adult tissues analyzed. Tks5β is a substrate for the Src tyrosine kinase and its expression is regulated through the proteasome degradation pathway. Together, these findings indicate the essentiality of the larger Tks5 isoform for correct mammalian development and the transcriptional complexity of the *sh3pxd2a* gene.

## Introduction

Genetic regulation of embryonic development is a complex process that underlies and, at the same time, is controlled by, specific molecular and cellular changes exquisitely coordinated in space and time. During the last 3 decades, the development of forward and reverse genetics through the use of model organisms such as *Drosophila melanogaster*, *Caenorhabditis elegans*, zebrafish and mouse have allowed the definition of key processes in embryo formation [1], [2], [3]. Importantly, these processes are evolutionarily conserved and have helped to elucidate the function of human orthologous genes. This has been particularly beneficial when these genes are involved in pathologies. In cancer, as one example, many developmentally important genes are reactivated during the epithelial-to-mesenchymal transition that precedes metastatic spread [4]. However, we do not yet fully understand the developmental regulation of the genome and its correlation to human disease. For instance, according to the Online Mendelian Inheritance in Man database (http://www.ncbi.nlm.nih.gov/omim, April 2014), there are 752 congenital diseases of Mendelian nature, or suspected to have a Mendelian basis, whose molecular cause is unknown. Although these diseases are rare in the population, there are more frequent inborn developmental errors of non-Mendelian inheritance where a polygenic origin is suspected but not determined [5], [6]. Another example is given by the clefts of the lip and the palate, where both genetic and environmental factors are involved [7], [8]. Overall, they represent a substantial number of congenital conditions whose genetics have not been elucidated and that as a whole represent a considerable clinical burden to society.

Tks5 is an adaptor protein first identified as a novel Src substrate [9]. Tks5 contains a phox homology (PX) domain located at the N-terminus and 5 SH3 domains interspersed with several polyproline motifs and 2 main Src phosphorylation sites (Y557 and Y619) [10]. Tks5 has been demonstrated to be necessary for the formation and function of the actin and protease rich-structures podosomes and invadopodia, collectively sometimes known as invadosomes [11]. Podosomes (in normal cells) and invadopodia (in cancer cells) are located on the ventral cellular surface where they establish close contact with the extracellular matrix and act in its degradation [12]. Src family kinases are crucial in podosome biology [13], [14], [15], [16], where they have been suggested to be involved in podosome initiation as well as disassembly [17]. In Src-transformed NIH3T3 cells, phosphorylation of Tks5 is one of the first events in podosome formation [18], after which Tks5 associates with Grb2 and binds to the phosphatidylinositol-3,4-bisphosphates that are forming at the membrane of the nascent podosome through its PX domain [12], [18], [19]. This binding facilitates the direct or indirect association with Nck1, Nck2 and N-WASP, and is followed by intense actin polymerization [10], [18]. In cancer cells, Tks5 is involved in invadopodia formation and function [11] and promotes reactive oxygen species generation by the NADPH oxidase system, by acting as an NADPH organizer that binds p22^phox^ [20], [21].

In humans, TKS5 is encoded by the *SH3PXD2A* gene, located on chromosome 10, with a size of 267 kb and a coding sequence formed by 15 exons. The closest homologue to TKS5 is the protein TKS4, encoded by the *SH3PXD2B* gene. *SH3PXD2A* and *SH3PXD2B* genes have a 47% sequence identity. They seem to have arisen from a common precursor gene since the tunicate *Ciona intestinalis* and the sea urchin *Strongylocentrotus purpuratus* present a single gene encoding a protein with a PX domain followed by 3 and 4 SH3 domains, respectively [22]. No homologue gene is found in flies or worms, indicating they appeared late in metazoan development. TKS4 has a PX domain followed by 4 SH3 domains, and each domain has an identity higher than 50% with respect to the corresponding TKS5 domains. TKS4 is also a Src substrate and essential for mature podosome formation and function [22]. Absence of TKS4 in humans and mice due to inactivating mutations or gene deletion leads to severe developmental defects in the craniofacial area, skeleton and heart as well as shortened lifespan, which are hallmarks of the Frank-Ter Haar syndrome [23]. Likewise, knock-down of Tks5 in zebrafish embryos causes abnormalities in neural crest cell-derived structures such as those on the craniofacial zone and pigment cells. Further analysis of zebrafish embryos and neural crest cells *in vitro* indicated that Src-activated Tks5 is necessary for proper neural crest cell migration [24]. In light of this finding, we wondered about the role of Tks5 in mammalian development, choosing the mouse as a model because of its genomic, physiological and pathological similarities to humans [25], [26], [27]. Here we describe the analysis of mice bearing an inactivating gene-trap insertion in intron 1 of the *sh3pxd2a* gene.

## Materials and Methods

### Ethics

This study was approved by the Sanford-Burnham Medical Research Institute Animal Care and Use Committee (protocols 07-070 and 10-052; IACUC assurance number A3053/01), and performed in accordance with the Institute of Laboratory Animal Research (NIH, Bethesda, MD) Guide for the care and Use of Laboratory Animals.

### Mice

*Sh3pxd2a* mutant mice were generated by gene-trapping by Lexicon Pharmaceuticals (The Woodlands, TX) (embryonic stem-cell clone ID OST445753). Inactivation of *sh3pxd2a* gene in these mice is due to insertion of the trapping vector VICTR 37 between exons 1 and 2 on chromosome 19. After quarantine, mice were maintained under standard pathogen-free conditions in a constant temperature and humidity environment with a 12∶12 light: dark cycle and *ad libitum* access to food and water at the Sanford-Burnham Medical Research Institute Animal Facility, La Jolla, California. The litters and adults were continuously monitored during daytime from Monday to Friday, and twice daily during daytime on Saturdays, Sundays, and holidays for signs of poor health. When pups/adults showed signs of poor health, such as shakiness and low activity with decreased body temperature, they were humanely euthanized. For neonates up to 7 days of age: hypothermia (placing the neonates in plastic bags on ice for 10 minutes) followed by decapitation with a razor blade. For animals older than 7 days: CO_2_ inhalation until mice stop breathing, followed by cervical dislocation. These methods have been approved by the Sanford-Burnham Medical Research Institute Animal Care and Use Committee following the American Veterinary Medical Association guidelines for the Euthanasia of Animals (https://www.avma.org/KB/Policies/Documents/euthanasia.pdf, sections S2.2.2.1, S2.2.2.3 and S2.2.4.2.2, and http://grants.nih.gov/grants/olaw/Euthanasia2007.pdf).

To assess disruption of the *sh3pxd2a* gene, embryonic fibroblasts were isolated from E12.5 embryos and genomic DNA and total protein were extracted and analyzed by PCR and immunoblotting, respectively. Mice carrying a homozygous trapping mutation are denominated Tks5^trap/trap^ or Tks5α/Tks5_long_ null mice, while heterozygous mice appear as Tks5^+/trap^ and wild types as Tks5^+/+^. *Sh3pxd2a* mutant mice were analyzed on a C57BL/6Jx129/SvJ mixed background, as provided by Lexicon Pharmaceuticals, and in a C57BL/6J pure background, which was obtained by backcrossing the mice for 7 generations. Strain purity was assessed by SNP analysis by the Murine Genetics Analysis Laboratory at U.C. Davis, CA. The phenotype of the *sh3pxd2a* mutant mouse has never been studied before. Homozygous mutant mice had three phenotypic manifestations: dying within the first 24 hours of life with presence of cleft palate; dying post day 1 of life without cleft palate; and a third group reaching adult life with no apparent phenotypic differences with respect normal littermates. We used 228 mice (n = 62 wild type, n = 113 heterozygous, n = 53 homozygous mutant) from a total of 30 litters on the C57BL/6Jx129/SvJ mixed background to determine accurately the percentage of mice with each of the three phenotypic manifestations observed when both alleles of *sh3pxd2a* are mutated. Fewer mice would have yielded a survival curve with significantly decreased survival probability of the homozygous mutant mice but would have allowed us neither to accurately determine the percentage of mutant mice in each phenotypic group, nor to have enough mice to properly study the phenotypes. For the C57BL/6J pure background we analyzed 120 mice (n = 24 wild type, n = 61 heterozygous, n = 35 homozygous mutant) from a total of 15 litters. We analyzed this quantity of mice to determine that the Tks5α mutation in a C57BL/6J pure background did not allow survival of mutant mice, in contrast to what happens in the C57BL/6Jx129/SvJ mixed background. Because at the outset we were naïve to the phenotypes we would observe, our animal use protocol included any survival situation as well as welfare for mice of any age.

Double Tks4/Tks5 mutant heterozygous mice were generated by crossing heterozygous *sh3pxd2a* mutant mice with heterozygous *sh3pxd2b* mutant mice [23]. Blastocysts were obtained as described [28] from 7 double heterozygous Tks4^+/−^, Tks5^+/trap^ pregnant females crossed with double heterozygous Tks4^+/−^, Tks5^+/trap^ males.

### Reagents

Puromycin, neomycin, PP2, SU6656 and MG132 were from EMD, blasticidin was from Invitrogen, and doxycycline was from Sigma. Epoxomicin and bortezomib were a gift from Dr. Dieter Wolf, Sanford Burnham Medical Research Institute, La Jolla, California. SU11333 [29] was synthesized by A. Singh in G. Roth laboratory at Sanford Burnham Medical Research Institute, Lake Nona, Florida. Saracatinib (AZD0530) was a gift from Astra Zeneca [30].

### Cell lines and culture conditions

Murine Embryonic Fibroblasts (MEFs) were isolated from mouse embryos (E12.5) as described [22]. NIH3T3 and 293T cells were obtained from the American Type Culture Collection. NIH3T3 clone 7 mouse fibroblasts expressing active chicken c-Src (SrcY527F) or the corresponding empty vector control cells have been previously described [9]. All cells were maintained in DMEM (Mediatech) containing 10% FBS (Hyclone). To generate Src-transformed MEFs, cells were infected with a retroviral pBabe construct encoding active human c-Src (Y530F) and selected with 10 µg/ml of puromycin (EMD). To generate lines expressing Tks5β, cells were infected with empty or Tks5β-expressing lentiviral pCDH constructs and selected with puromycin or neomycin. To generate Src-transformed cells expressing Tks5_short_, cells were infected with lentiviral stocks generated with a pCW22-Tks5_short_ construct obtained from Dr. Tyler Jacks [31]. Cells were selected with 5 µg/ml blasticidin. To induce the expression of Tks5_short_, cells were treated with 50 nM doxycycline for 24 h.

### 5′ Rapid Amplification of cDNA ends (RACE)

Identification of the new transcription initiation site on the *sh3pxd2a* gene was done by 5′ RACE (SMART RACE cDNA Amplification kit; Clontech) analysis of Src-transformed and non-transformed NIH3T3 cells total RNA, according to manufacturer's instructions, with the following exceptions: antisense primers were located on exon 14 (5′-GCCTTCTGTCCGCCTCGAA-3′) or exon 15 (5′-CCTCGGCCTCCTTAGGCTTG-3′), and the PCR amplification step was performed using a sense primer (5′-AGCCCCTGCCAGTCCTAACC-3′) located upstream of the putative alternative ATG on intron V. The resulting products were cloned into the pCR-XL-TOPO (Invitrogen) vector, digested with EcoRI and sequenced.

### Cloning

To generate a lentiviral vector construct encoding mouse Tks5β, a XhoI/BlpI fragment was excised from the 5′RACE product cloned into pCR-XL-TOPO vector and cloned in frame into a XhoI/BlpI-digested pCDNA3 vector expressing full length Tks5 (not containing exons 7 or 10). A XhoI/XbaI fragment from the resulting construct was then cloned into a XhoI/XbaI-digested pCDH-MCS-EF1-puro lentiviral vector (System Biosciences).

### RNA isolation and qPCR analysis

Total RNA from cells and tissues was purified using the TRIzol reagent (Invitrogen) according to manufacturer's instructions. We used TissueRuptor (Qiagen) homogenizer for tissue disruption. 1 µg RNA was treated with Deoxyribonuclease I (Invitrogen) prior to cDNA generation using the SuperScript III first-Strand Synthesis System kit (Invitrogen). To quantify the amount of mRNA of Tks5α and Tks5β on murine tissues, the following primers were designed: for Tks5α sense 5′-GGAGGACCCCTCTAAACAC-3′ (exon 1); antisense 5′-ATCCTTTGCTTCGGATCCTT-3′ (exon 3); and for Tks5β sense 5′-TGTCTATCTGTTGTTGTTTCTTTTTC-3′ (Tks5β unique sequence on exon 6β); antisense 5′-GACCCAACCTTGCTCTTCAG-3′ (exon 9). Total actin expression was used as reference gene and was detected with the following primers: sense 5′- TGTTACCAACTGGGACGACA-3′ (exon 3); antisense 5′-GGGGTGTTGAAGGTCTCAAA-3′ (exon 4). All products were sequenced to assess their specificity. Quantitative PCR were performed on an MX3000 real-time PCR thermal cycler (Stratagene). Results were analyzed and plotted according to the following equation: Result  =  [2 ^- (Ct Tks5 – Ct total actin)^] ×10,000. Results were multiplied by 10,000 for plotting purposes.

### Immunoprecipitation and immunoblotting

Cells were lysed in ice-cold lysis buffer containing 50 mM Tris pH 7.6, 150 mM NaCl,1% TritonX-100, 10 mM NaF, 100 µM sodium orthovanadate and protease inhibitors (Roche). Protein concentration was measured using the BCA assay (Fisher) and the same amount of total protein (between 0.5 and 1 mg) was used to immunoprecipitate Tks5. Tks5 antibodies used in this study include: 1) an in-house polyclonal Tks5 antibody (named 1737), raised against the fourth SH3 domain of Tks5 (used for immunoprecipitation and immunoblotting), 2) an in-house polyclonal antibody raised against the PX domain (named 7768), used for immunoblotting, c) an in-house monoclonal Tks5 antibody (named 6G1) raised against the fourth SH3 domain of Tks5 (used for immunoprecipitation), and 4) a commercial polyclonal Tks5 antibody raised against the fourth SH3 domain (Millipore catalog number 09-268), used for immunoblotting. Complexes were captured with protein A-conjugated beads (EMD) for 2 h at 4°C. After electrophoresis and electrotransfer, membranes were blotted with 1737 or anti-pTyr antibody (4G10, Millipore). Secondary antibodies conjugated with Alexa Fluor 680 were from Life Sciences Technology, and IR800 antibodies were from Rockland. Bands were visualized and quantified using an Odyssey infrared imaging system (LI-COR).

### Phosphatase treatment

Cell lysates were immunoprecipitated for Tks5 using 6G1 antibody and lambda protein phosphatase treatment (Cell Signaling Technologies) was performed after the last immunoprecipitation wash. Phosphatase was added according with the manufacturer's instructions in the presence or absence of 100 µM of sodium orthovanadate. Samples were boiled in sample buffer and separated by electrophoresis.

### Statistics

Data were analyzed with the Graph Pad Prism 5 software. Mice survival data were analyzed using Mantel-Cox and Gehan-Breslow-Wilcoxon statistical tests. For other experiments results are represented as mean value and standard error of at least three replicates. Statistical significance was calculated using the Student's t test.

## Results

### Homozygous mutation of *sh3pxd2a* in mice causes cleft palate

**Figure 1:**
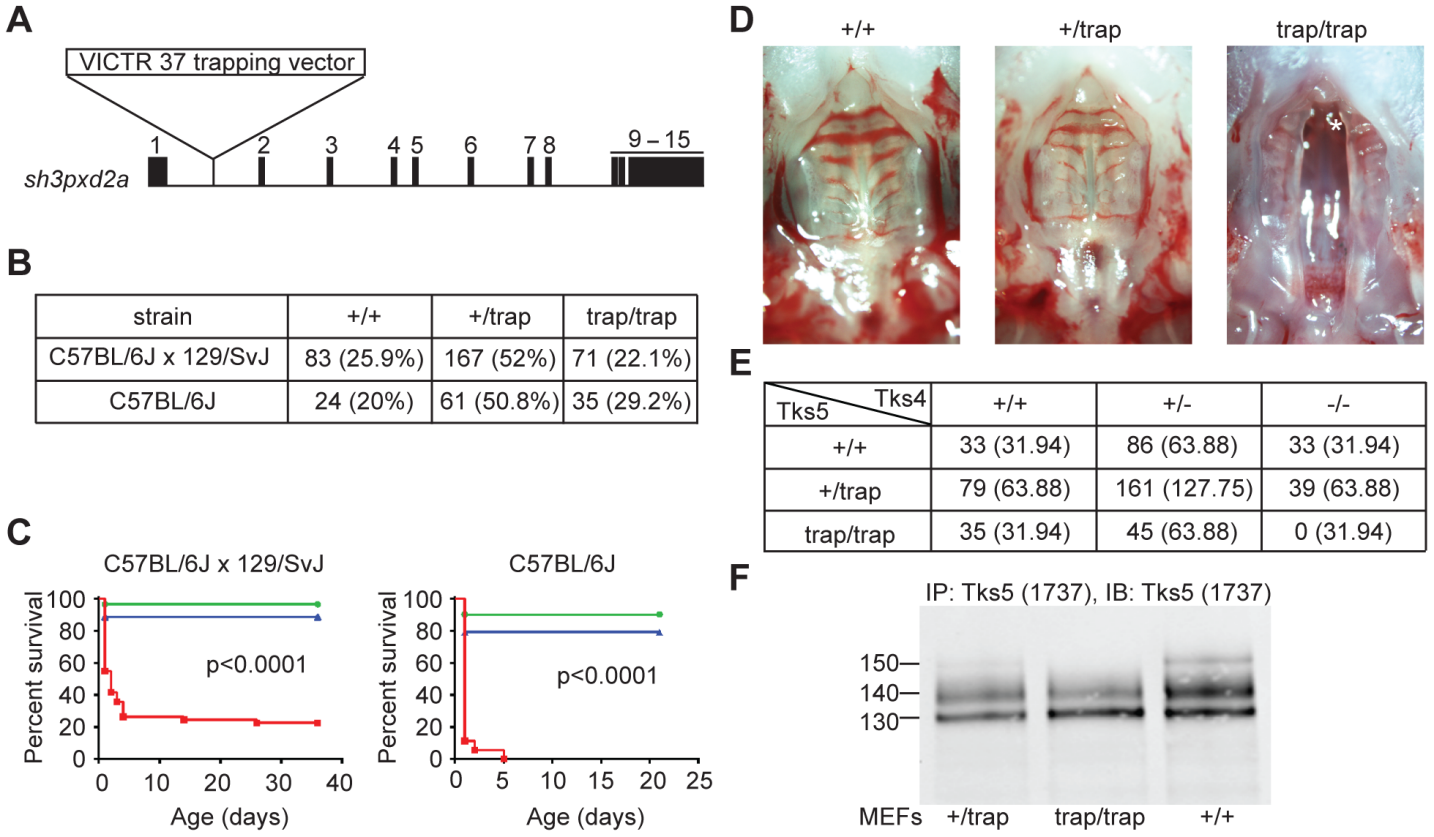
The *sh2pxd2a* gene is essential for correct mammalian development. (A) Map of the *sh2pxd2a* gene with location of the trapping cassette used to generate the *sh2pxd2a* mutant mice. Exons are indicated as black boxes with order number on top. Trapping cassette is inserted in intron 1. (B) Absolute numbers and percentages (in parentheses) of *sh3pxd2a* mice (Tks5) born from heterozygous intercrosses in a C57BL/6Jx129/SvJ mixed strain background or C57BL/6J pure strain. (C) Survival curves of Tks5 mice in a C57BL/6Jx129/SvJ mixed background (left) and a pure C57BL/6J background (right). Wild type mice are indicated in blue, Tks5^+/trap^ mice in green and Tks5^trap/trap^ mice in red. Statistical analysis was conducted using both Mantel-Cox and Gehan-Breslow-Wilcoxon tests, with p values of less than 0.0001 in all cases. (D) Representative photographs showing the palates of Tks5 wild-type (+/+), Tks5 heterozygous mutant (+/trap) and Tks5 homozygous mutant (trap/trap) neonates. Asterisk denotes the nasal septum. (E) Offspring genotype numbers born from Tks4^+/−^, Tks5^+/trap^ double heterozygous mice intercrosses. Numbers outside parentheses show the observed number of mice born for each indicated genotype, while the numbers in parentheses correspond to the expected number of mice according to Mendelian laws. (F) Immunoblot for Tks5 in protein extracts from MEFs obtained from Tks5 wild type, heterozygous (+/trap) and homozygous mutant (trap/trap) E12.5 mouse embryos. Tks5 was immunoprecipitated and immunoblotted with a polyclonal Tks5 antibody raised against the 4^th^ SH3 domain (1737).

In order to study the function of Tks5 in mammalian development, we obtained *sh3pxd2a* gene-trapped mice from Lexicon Pharmaceuticals. The trapping cassette (vector VICTR37) was inserted on intron 1 of the *sh3pxd2a* gene on chromosome 19 (Figure 1 A). *Sh3pxd2a* homozygous mutant mice (Tks5^trap/trap^ hereafter) were born at Mendelian ratios (Figure 1B) and had reduced survival with respect to wild-type and heterozygous littermates (Figure 1 C). On a C57BL/6Jx129/SvJ mixed genetic background, 50 percent of the Tks5^trap/trap^ mice died within the first 24 hours of life, 30 percent died between day 1 of life and time of weaning, and the remaining 20 percent survived throughout adulthood with no apparent phenotypic defects. Tks5^trap/trap^ pups dead within the first 24 hours of life did not have milk in their stomachs, and when analyzed all of them presented complete cleft of the secondary palate (Figure 1 D). Gross analysis of these dead pups at age ≤24 hours showed that Tks5^trap/trap^ neonates had sporadic bifid xiphisternum (data not shown), but no other obvious developmental defects were found. Tks5^trap/trap^ pups dying between day 1 of life and weaning time were able to eat and none of them presented cleft palate, but eventually they failed to thrive and died. To elucidate if the phenotypic diversity in the Tks5^trap/trap^ mice was due to irregular efficiency of the trapping cassette or to genetic background impurity, *sh3pxd2a* mice were backcrossed into the C57BL/6J pure background. Tks5^trap/trap^ mice in a pure C57BL/6J background were also born at Mendelian ratios (Figure 1 B). However, presence of complete cleft of the secondary palate associated with neonatal death of Tks5^trap/trap^ mice increased to 90 percent. The 10 percent remaining died shortly thereafter of unknown causes, but without presence of cleft palate (Figure 1 C). Therefore, strain purity can affect the phenotypic manifestations of *sh3pxd2a* gene mutation. We did not find any gross phenotypic differences between heterozygous and wild-type mice, or between Tks5^trap/trap^ male and female mice, either in the mixed or in the pure C57BL/6J background.

We have previously cloned and characterized Tks4, a related adaptor protein that presents the highest homology to Tks5 [22]. Both Tks4 and Tks5 are necessary for mature podosome formation [11] but they have distinct functions, since Tks4 null Src-transformed murine embryonic fibroblasts (MEFs) transfected with human TKS5 protein recover the ability to form podosomes but they fail to degrade gelatin, while transfection with human TKS4 rescues both podosome formation and function [22]. Tks4 null mice are born at Mendelian ratios but present severe developmental anomalies in the craniofacial bones, skeleton, heart and eyes that compromise their long-term survival [23]. To find out if Tks5 and Tks4 interact during mammalian development, we created mice doubly heterozygous for Tks4 and Tks5 by crossing *sh3pxd2a* and *sh3pxd2b* mutant mouse lines (both in a C57BL/6Jx129/SvJ mixed background), and used them in intercross matings to analyze the offspring at birth. Figure 1E shows the number of mice born for each expected genotype. No Tks4^-/-^; Tks5^trap/trap^ double null mice were born in a total of 511 mice from 67 litters analyzed, suggesting an overlapping and/or compensatory function of Tks4 and Tks5 during development. The numbers of mice homozygous mutant for one gene and heterozygous for the other (Tks4^-/-^; Tks5^+/trap^ and Tks4^+/−^; Tks5^trap/trap^) were below the expected Mendelian ratios as well, supporting this hypothesis.

Recently, it has been shown that trophoblasts can form podosomes [32], which suggests a role for the podosome regulators Tks4 and/or Tks5 in trophoblast function and therefore in implantation. To investigate if the absence of both Tks4 and Tks5 proteins caused lethality before or after implantation, we genotyped E3.5 pre-implantation blastocysts from double heterozygous (Tks4^+/−^; Tks5^+/trap^) mice intercross matings. We detected 11 Tks4^-/-^; Tks5^trap/trap^ double null blastocysts out of a total of 116 blastocysts analyzed, indicating that absence of Tks proteins does not cause embryonic lethality before implantation. Together, these data demonstrate the importance of Tks5 for correct mammalian development post-implantation, and suggests an intricate regulation of Tks proteins during implantation and/or embryonic development, with distinct roles at some developmental stages and compensatory/redundant functions at others.

### Different isoforms of Tks5 are generated by alternative transcription of the *sh3pxd2a* gene

**Figure 2:**
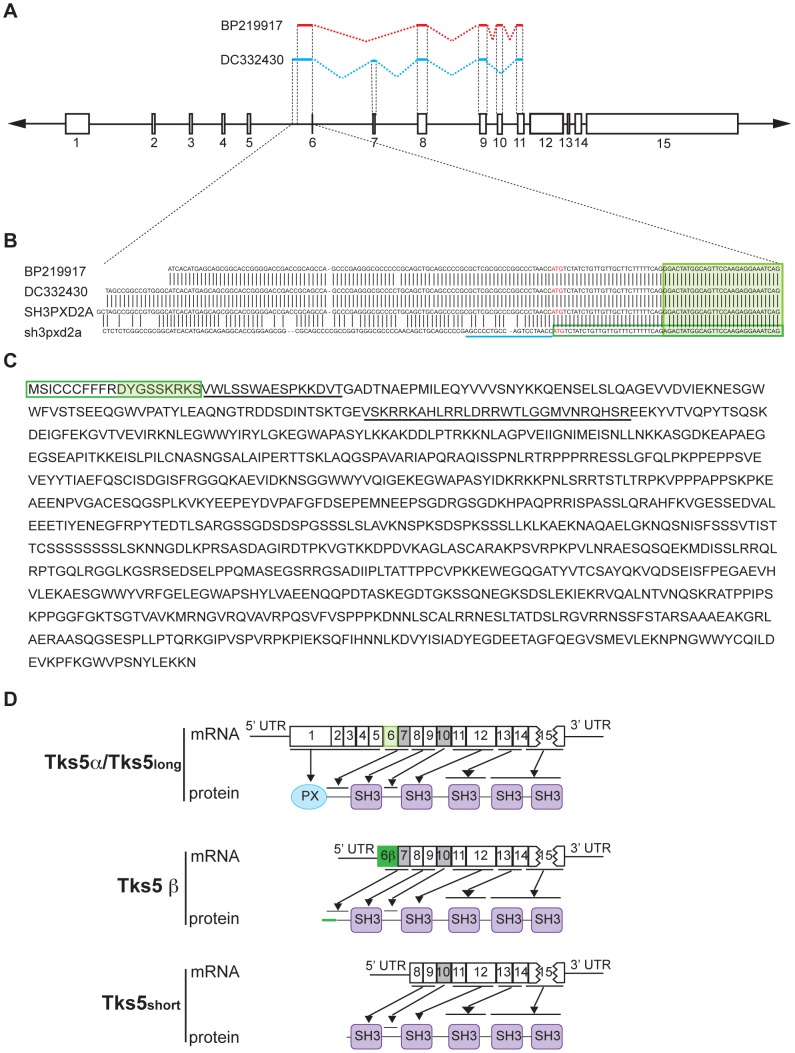
The *sh3pxd2a* gene encodes for different isoforms of Tks5. (A) Alignment of human ESTs DC332430 (blue) and BP219917 (red) with the human *SH3PXD2A* gene (bottom). *SH3PXD2A* exons are represented by boxes and lines with the exon number at the bottom. Exons included in each EST are indicated by a colored continuous line on top of the corresponding exon framed between dashed black lines. Dashed colored lines indicate splicing. (B) Sequence of the 5′ ends of the ESTs BP219917 and DC332430 up to the end of exon 6 and alignment to the sequences of human *SH3PXD2A* and mouse *sh3pxd2a* genes. Base coincidence is indicated by black vertical lines. Exon 6 among sequences is shown inside a light green filled box, blue line indicates binding site of the 5′ RACE primer, red ATG bases show the alternative initiation of translation codon site and exon 6β is denoted by a dark green-lined box. (C) Predicted amino acid sequence of the new Tks5β isoform. Residues encoded by exon 6β are inside a dark green-lined box, with the ones encoded by exon 6 shown in a light-green background. Underlined residues indicate those encoded by exon 7 and 10, which may or may not be present in the protein due to alternative splicing. (D) Schematic representation of the 3 transcripts that can arise from alternative transcription of the mouse *sh3pxd2a* gene and their correspondence to the different protein domains. Exons are represented by boxes with the corresponding number inside, exon 6 is represented by a light-green box and exon 6β by a dark-green box. Exons 7 and 10 are represented by gray boxes since they are subject to alternative splicing and may or may not be part of the transcript. Black lines and arrows show the correspondence between exons and protein domains. Tks5_short_ and Tks5_long_ isoforms have been described recently by Li *et al*. [31], where Tks5_long_ is identical to the Tks5α described here.

To analyze the degree of protein loss achieved by the trapping cassette, we isolated murine embryonic fibroblasts (MEFs) from E12.5 embryos from Tks5^+/trap^ intercrossings and analyzed Tks5 protein expression by immunoblot (Figure 1 F). Tks5 protein detection by immunoblot has been characterized by the presence of a triplet set of bands ranging from 130–150 kDa, with the exact origin and characteristics of each being unknown [9]. Surprisingly, we found that the presence of the trapping cassette affected only the expression of the top band of the Tks5 triplet, which was diminished in the heterozygous MEFs and absent in homozygous MEFs. This finding made us hypothesize about the presence of an internal alternative promoter downstream of intron I that would generate shorter forms of Tks5 unaffected by the action of the trapping cassette. Supporting this hypothesis, we found two human EST sequences (GenBank accession numbers DC332430 and BP219917) with homology to the human *SH3PXD2A* gene coding sequence and that lack exons 1 to 5, which are the ones encoding the PX domain (Figure 2 A) [9]. Instead, they have a 29-base sequence upstream the beginning of exon 6 that starts with an in-frame ATG (Figure 2 B). Also, DC332430 contains exon 7 but lacks exon 10, while BP219917 lacks exon 7 but contains exon 10: these two exons have been described previously to be alternatively spliced [9]. Alignment of the human EST sequences to the mouse *sh3pxd2a* gene sequence revealed a high homology and the existence of the same in-frame ATG (Figure 2 B). To ascertain if this conserved ATG was indicating a new alternative translational start site of the *sh3pxd2a* gene, we performed 5′-RACE in cDNA from Src-transformed and non Src-transformed NIH3T3 mouse fibroblasts, with forward primer located upstream the putative start site (Figure 2 B) and reverse primers located either in exon 14 or 15. We obtained three clones from Src-transformed NIH3T3 cells and one clone from non Src-transformed NIH3T3 cells which encoded the same sequence, starting at the alternative ATG on intron V and lacking exon 7 but containing exon 10 (Figure 2 C). This finding suggests the existence of an alternative promoter in intron V that can encode a distinct isoform of Tks5 lacking the PX domain, since it would not contain exons 1 to 5. The new isoform coding sequence starts 29 bp upstream of exon 6 and includes it as part of one alternative first exon 58 bp long that we named exon 6β (Figure 2 C and 2 D).

**Figure 3:**
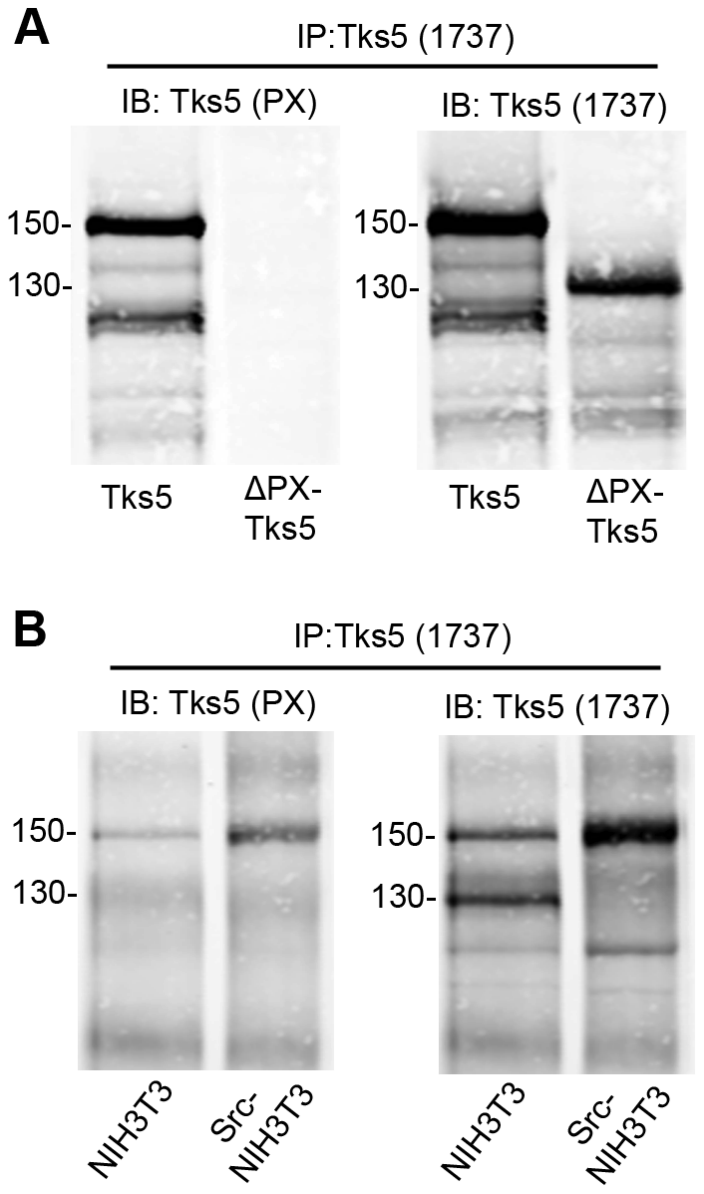
Immunoblot detection of PX-lacking and containing Tks5 isoforms. (A) Lysates from 293T cells transfected with constructs expressing full-length Tks5 protein (Tks5) or Tks5 protein lacking the PX domain (ΔPX Tks5) were immunoprecipitated with a polyclonal Tks5 antibody raised against the 4^th^ SH3 domain (1737) and sequentially immunoblotted with a Tks5 PX antibody (left panel) or the 1737 antibody (right panel). (B) Lysates from parental or Src-transformed NIH3T3 cells were immunoprecipitated with a polyclonal Tks5 antibody raised against the 4^th^ SH3 domain (1737) and sequentially immunoblotted with a Tks5 PX antibody (left panel) or the 1737 antibody (right panel). Numbers on the left indicate kDa.

The existence of different alternative transcriptional start sites in the *sh3pxd2a* gene might explain the detection of several bands when immunoblotting for Tks5, with the highest band (150 kDa) corresponding to the protein with the PX domain, and the lower ones (140 and 130 kDa) lacking the PX domain. To test this possibility, we generated a rabbit polyclonal antibody against the PX domain of Tks5, which should not recognize a PX-lacking Tks5 isoform. We tested the specificity of the Tks5 PX antibody on 293T cells transfected with constructs encoding full-length Tks5 or a Tks5 mutant lacking the PX domain (ΔPX Tks5) (Figure 3 A). We immunoprecipitated the 293T lysates with the 1737 antibody (raised against the 4^th^ SH domain of Tks5), then sequentially immunoblotted the same membrane with the Tks5 PX and 1737 antibodies. As expected, the PX domain antibody detected only full length Tks5 and not the Tks5 construct lacking the PX domain (Figure 3 A, left panel). To ascertain whether the triplet we observe in NIH3T3 mouse fibroblasts corresponds to PX-containing and PX-lacking Tks5 isoforms, we immunoprecipitated Src-transformed and parental NIH3T3 lysates with 1737 and sequentially immunoblotted the same membrane with the Tks5 PX and 1737 antibodies (Figure 3 B). In agreement with our hypothesis, we observed that only the upper band (150 kDa) of both cell lines was recognized by the Tks5 PX antibody, whereas all Tks5 bands were recognized by the 1737 antibody. These results confirm that the 150 kDa band of the Tks5 triplet corresponds to a PX domain-containing Tks5 isoform and the lowest bands at 140 and 130 kDa to Tks5 isoforms lacking the PX domain. We named Tks5α the PX domain-containing isoform, and Tks5β the isoform with a coding sequence starting on exon 6β and lacking the PX domain (Figure 2 D). Recently, an additional isoform of mouse Tks5, named Tks5_short_, has been described by Li et al. [31]. Tks5_short_ contains exons 8 to 15 preceded by a unique sequence encoded in intron VII (Figure 2 D). Li et al. describe as well the PX-domain containing Tks5 isoform (that they named Tks5_long_), which corresponds to the Tks5α isoform described here. Collectively, these data strongly suggest that the *sh3pxd2a* gene gives rise to different mRNA transcripts that are translated into several Tks5 isoforms: a PX-containing Tks5α/Tks5_long_ isoform with a site of initiation of transcription upstream of exon 1, and the PX-lacking isoforms Tks5_short_ and the novel Tks5β, both having a site of initiation of transcription downstream of exon 5.

### Tks5α and Tks5β mRNA tissue expression analysis reveals an isoform cross-regulation during normal physiological conditions

**Figure 4:**
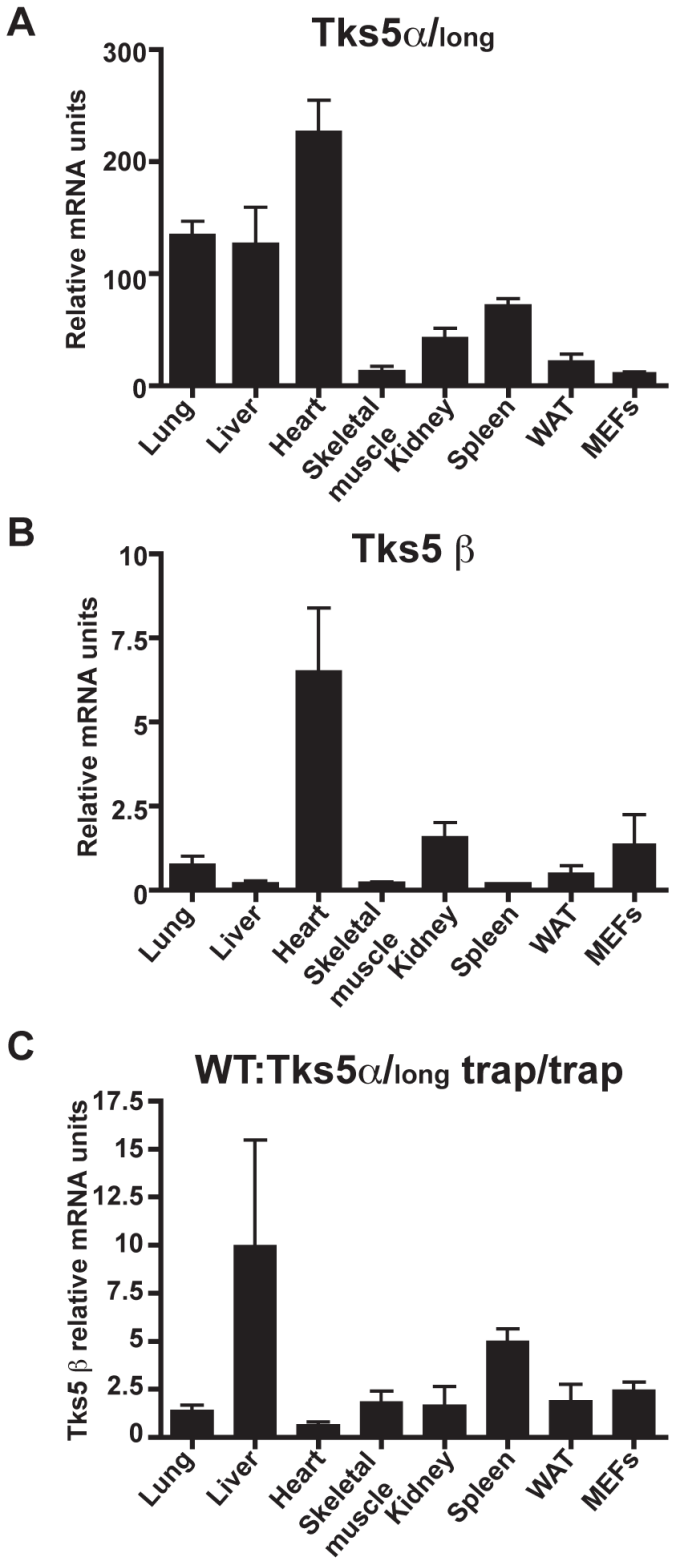
Tks5α/Tks5_long_ and Tks5β mRNA expression in adult mice organs during normal physiological conditions. Total RNA was extracted from organs from 3 wild type mice aged 5–8 months (A–C) and 3 Tks5α/Tks5_long_ null littermates (C), and retrotranscribed into cDNA for Tks5α/Tks5_long_ and Tks5β specific mRNA detection by qPCR. (A) Tks5α/Tks5_long_ and (B) Tks5β mRNA detection in wild-type murine organs and MEFs. The amount of Tks5α/Tks5_long_ or Tks5β was corrected by the expression of total actin mRNA in that same sample (see materials and methods). Columns show the average of 3 different wild type mice and 2 different wild type MEF cell lines. (C) Tks5β mRNA expression ratio between wild type (WT) and Tks5α/Tks5_long_ null organs and MEFs. Columns show the average of 3 ratios corresponding to 3 pairs wt-trap littermates, and for MEFs show the average of 5 ratios corresponding to 2 null lines: 1 wt line obtained from one litter, and 3 null lines: 1 wt obtained from a second litter. WAT: white adipose tissue.

Next, we analyzed the expression of Tks5α/Tks5_long_ and Tks5β mRNA in different murine organs by RT-qPCR (Figure 4). To specifically detect the mRNA of the Tks5α isoform we designed qPCR primers aligning to exons 1 and 3, which encode for part of the PX domain. To specifically detect the Tks5β isoform, we designed primers located in the newly discovered unique sequence of exon 6β and in exon 9. We normalized the results against the total actin mRNA expression in each organ analyzed. We detected expression of both isoforms in all organs analyzed (lung, liver, heart, skeletal muscle, kidney, spleen and white adipose tissue) and in MEFs (Figure 4 A and B), but the expression of the Tks5α/Tks5_long_ mRNA isoform was markedly higher in all tissues compared to the Tks5β isoform, ranging from over 400-fold in liver and spleen to 27-fold in kidney and 8-fold in MEFs. Heart was the organ with the highest expression of both isoforms compared to total actin expression. Lung and liver also had a high expression of the Tks5α/Tks5_long_ isoform. Next, we wondered if expression of Tks5α/Tks5_long_ could affect the Tks5β mRNA expression and vice versa. To assess this, we took advantage of the fact that 20% of the Tks5α/Tks5_long_ null mice in the C57BL/6Jx129/SvJ mixed background survive into adulthood (Figure 1 C), and compared the expression of the Tks5β mRNA isoform in organs from 3 of these mice and as many wild-type littermates (5–8 months old) (Figure 4 C). We compared as well the Tks5β mRNA expression in Tks5α/Tks5_long_ mutant and wild type MEFs (passage 1). First, we could unequivocally detect expression of Tks5β mRNA in all tissues and MEFs from Tks5α null mice, while, as expected, we detected extremely low amounts or no expression of Tks5α/Tks5_long_ mRNA (data not shown). Then, we compared the Tks5β mRNA expression between wild type mice and Tks5α/Tks5_long_ mutant littermates. We observed that the relative abundance of Tks5β in lung, skeletal muscle, kidney, white adipose tissue and MEFs of wild-type mice is just slightly elevated with respect Tks5α/Tks5_long_ mutant mice (1-fold to 3-fold). However, liver and spleen of wild-type mice have a 10-fold and 5-fold higher expression of Tks5β with respect to Tks5α/Tks5_long_ mutant mice. In contrast, hearts of wild-type mice have a 50% decreased expression of Tks5β mRNA in comparison to null mice. These data indicate that both Tks5α/Tks5_long_ and Tks5β isoforms are expressed at the transcript level in most adult organs during normal physiological conditions, and suggest a differential expression of Tks5α/Tks5_long_ and Tks5β isoforms in an organ-dependent manner. Furthermore, comparison of Tks5β mRNA expression between wild-type mice and littermates lacking Tks5α/Tks5_long_ also suggest cross-regulation of mRNA abundance between both isoforms.

### Protein expression of the endogenous non PX-containing Tks5 isoforms is regulated by the activity of Src

**Figure 5:**
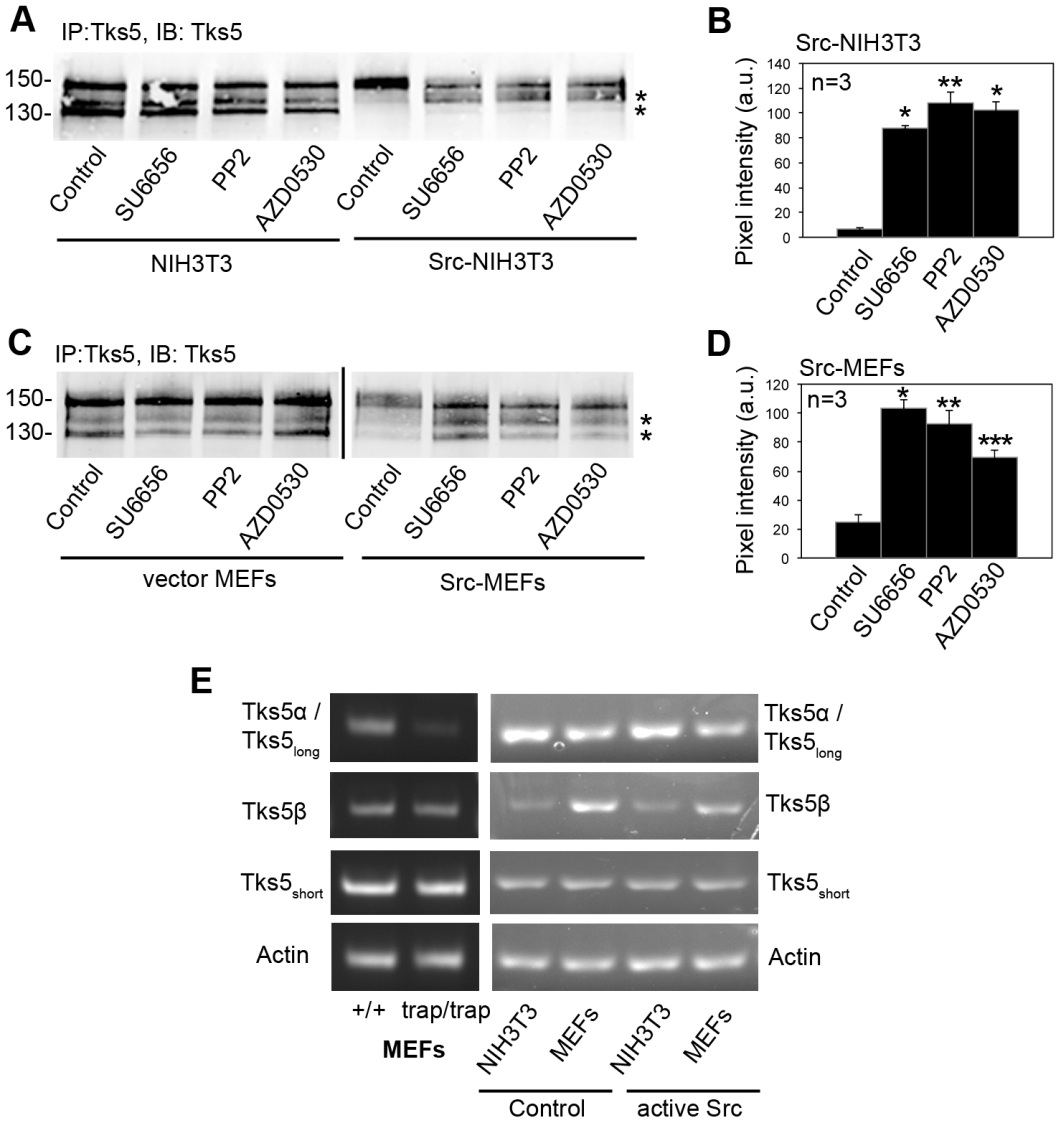
Protein expression of the endogenous non PX-containing Tks5 isoforms is regulated by the activity of Src. (A) Tks5 immunoblotting of extracts from Src-transformed NIH3T3 cells and their control counterparts upon treatment with a 5 µM concentration of the indicated Src family kinase inhibitors for 24 h. Numbers on the left indicate kDa. Asterisks indicate Tks5 middle and low bands corresponding to non-PX containing Tks5 isoforms. (B) Quantification of three independent experiments using densitometric analysis for the 140 and 130 kDa bands combined. Average pixel intensity (arbitrary units) and standard deviations are shown. (*) p<0.0001; (**) p<0.0005 using Student's t test. (C) Tks5 immunoblotting of extracts from Src-transformed MEFs and their control counterparts upon treatment with a 5 µM concentration of the indicated Src family kinase inhibitors for 24 h. Numbers on the left indicate kDa. Asterisks indicate Tks5 middle and low bands corresponding to non-PX containing Tks5 isoforms. (D) Quantification of three independent experiments using densitometric analysis for the 140 and 130 kDa bands combined. Average pixel intensity (arbitrary units) and standard deviations are shown. (*) p<0.0001; (**) p<0.01; (***) p<0.001 using Student's t test. (E) RT-PCR analysis for the indicated Tks5 isoforms. Left column: Analysis on MEFs from wild type and Tks5α/Tks5_long_ null mice. Right column: Analysis on control or Src-transformed cells (MEFs or NIH3T3) as indicated.

Transformation of NIH3T3 mouse fibroblasts by active Src induced an increase in the levels of the highest molecular weight Tks5 band (150 KDa), concomitant with the disappearance of the two Tks5 bands at 140 and 130 kDa, which correspond to PX-lacking Tks5 forms (Figures 3 B, 5 A and [11]). This effect was not cell-line specific, since transformation of primary MEFs with active Src had the same effect (Figure 5 C compare lanes 1 and 5). Furthermore, Src kinase activity was necessary to mediate the switch between higher and lower molecular weight bands, as evidenced by treatment with the pharmacological inhibitors of Src-family kinases SU6656, PP2 and AZD0530 (saracatinib) in Src-transformed cells (Figure 5 A–D). Interestingly, treatment with Src-family kinase inhibitors significantly increased the expression of the PX-lacking Tks5 isoforms in Src-transformed NIH3T3 (Figure 5 A, asterisks) and MEFs (Figure 5 C, asterisks), as quantified in Figure 5B and D. We wondered whether this effect was due to differences in the transcription of the PX-containing and PX-lacking Tks5 isoforms between control and Src-transformed cells. To address this, we analyzed the effect of Src transformation on the mRNA expression of the different Tks5 isoforms by RT-PCR. We used Tks5α/Tks5_long_ and Tks5β specific primers (Figure 4), along with primers specific for the Tks5_short_ form obtained from Dr. Tyler Jacks [31], to perform PCR. As expected, in primary MEFs derived from Tks5^trap/trap^ mice, only the mRNA for the Tks5α/Tks5_long_ was significantly decreased, whereas the mRNA expression for the other two non PX-containing Tks5 isoforms (Tks5β and Tks5_short_) was unchanged (Figure 5 E, left panel). Next, we performed RT-PCR analysis to detect the mRNA for the different Tks5 isoforms in NIH3T3 fibroblasts or primary MEFs along with their Src-transformed counterparts. No significant changes in the mRNA levels for any of the Tks5 isoforms were observed among control and Src-transformed cells (Figure 5 E, right panel), indicating that the changes previously observed in the protein expression of the Tks5 isoforms upon Src transformation are likely due to post-translational modifications.

### Tks5β protein expression is regulated by Src activity and the proteasome degradation pathway

**Figure 6:**
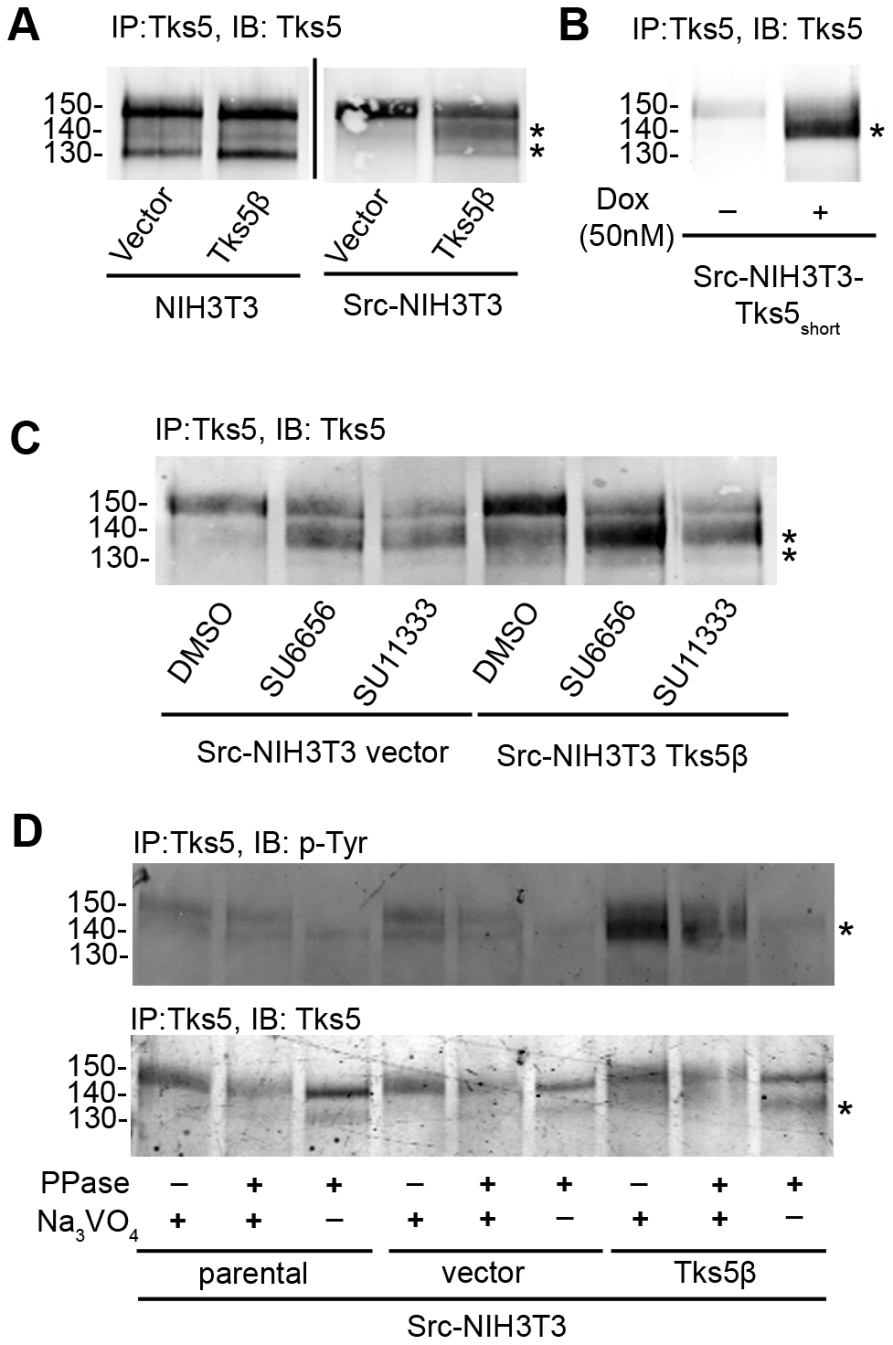
Tks5β protein expression is regulated by Src activity. (A) Tks5 immunoblotting of extracts from Src-transformed NIH3T3 cells and their control counterparts expressing empty vector or Tks5β. Number on the left indicate kDa. Asterisks indicate Tks5 middle (140 kDa) and low (130 kDa) bands. (B) Tks5 immunoblotting on extracts from Src-transformed NIH3T3 cells expressing an inducible Tks5_short_ isoform with or without induction with Doxycycline for 24 h. Number on the left indicates kDa. Asterisks indicate Tks5 middle (140 kDa). (C) Tks5 immunoblotting on extracts from Src-transformed NIH3T3 cells expressing empty vector or Tks5β upon treatment with a 5 µM concentration of the indicated Src family kinase inhibitors for 16h. Number on the left indicates kDa. Asterisks indicate Tks5 middle and low bands corresponding to non-PX containing Tks5 isoforms. (D) Tks5 immunoblotting on extracts from Src-transformed NIH3T3 cells untreated or expressing empty vector or Tks5β upon treatment with lambda protein phosphatase in the presence or absence of sodium orthovanadate. Asterisk indicate Tks5 middle (140 kDa) band.

To verify that our 5′-RACE product (Figure 2 C) encodes a Tks5 protein of the expected molecular size, we used the 5′-RACE product to generate a lentiviral-based expression construct for Tks5β. We generated lentiviruses and infected parental and Src-transformed NIH3T3 mouse fibroblasts and selected the infected cells with puromycin to generate stable cell lines. Protein extracts from the resulting cell lines were analyzed by immunoprecipitation with Tks5 6G1 antibody followed by immunoblotting with a polyclonal commercial Tks5 antibody. Expression of Tks5β in NIH3T3 cells rendered a single band at 130 kDa, whereas expression in Src-transformed mouse fibroblasts rendered two bands at 140 and 130 kDa that exhibited identical migration patterns to the Tks5 lower molecular weight bands in untransformed mouse fibroblasts (Figure 6 A). This was in contrast with the overexpression of the Tks5_short_ isoform, which rendered a single “middle” band at 140 kDa (Figure 6 B) in Src-transformed NIH3T3 cells. These results confirmed that our 5′-RACE product for Tks5β encodes for a Tks5 protein of the expected molecular size.

We consistently observed across independent infections that the expression of Tks5β was lower in Src-transformed fibroblasts than in parental cells (Figure 6 A). This suggested that Tks5β might be unstable in the presence of active Src, in a fashion similar to the endogenous non-PX containing Tks5 bands (Figure 5 A, C). To address that question, we treated control and Tks5β expressing Src-transformed cells with the Src family kinase inhibitors SU6656 and its derivative SU11333, and analyzed the effect on the expression of Tks5. Consistent with the effect of inhibiting Src activity on the protein expression of the endogenous non-PX containing Tks5, inhibition of Src in Tks5β-expressing Src-transformed fibroblasts increased the amount of the middle and lower Tks5 bands (Figure 6 C). These results confirmed that the Tks5β protein is unstable in cells with elevated Src kinase activity.

Tks5 was identified as a direct Src substrate [9]. Two main tyrosine residues in Tks5 (Tyr557 and Tyr619) are targets of Src kinase activity [9], [10]. These tyrosine residues are present in both Tks5α/Tks5_long_ and Tks5β. To analyze whether Tks5β is tyrosine-phosphorylated in Src-transformed NIH3T3 cells, we subjected cell extracts from parental, empty vector control and Tks5β-expressing Src-transformed NIH3T3 fibroblasts to treatment with lambda phosphatase in the presence or absence of the phosphatase inhibitor sodium orthovanadate (Figure 6 D). After de-phosphorylation, samples were separated by electrophoresis, transferred and sequentially immunoblotted with an anti-phosphotyrosine antibody and a Tks5 antibody. In parental and empty vector control cells, phosphatase treatment in the absence of sodium orthovanadate led to undetectable tyrosine phosphorylation of the Tks5α/Tks5_long_ form, along with the appearance of a smaller Tks5 form whose mobility is compatible with that of the middle 140 kDa band (Figure 6 D). This would be consistent with the appearance of the non-PX containing forms of Tks5 in Src-transformed cells after treatment with Src kinase inhibitors (Figure 5 A, C). Interestingly, the small amount of exogenous Tks5β present in Src-transformed extracts showed high levels of tyrosine phosphorylation, and the inhibition of this phosphorylation by phosphatase treatment in the absence of sodium orthovanadate led to the stabilization of the overexpressed Tks5β (Figure 6 D). Taken together, these results indicate that tyrosine phosphorylation of Tks5β regulates its protein stability.

**Figure 7:**
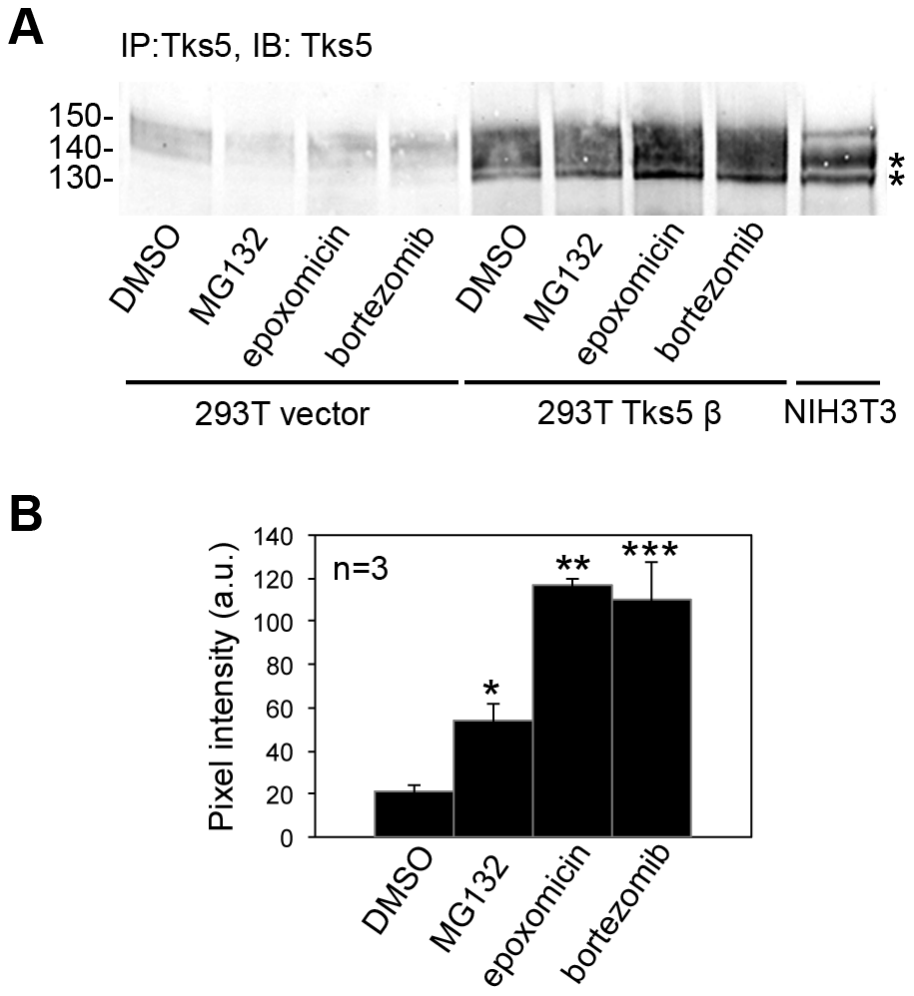
Tks5β protein abundance is regulated by the proteasome degradation pathway. (A) Tks5 immunoblotting on extracts from 293T cells expressing empty vector or Tks5β upon treatment with the proteasome inhibitors MG132 (5 µM), epoxomicin (50 nM) or bortezomib (50 nM) for 16 h. An extract of NIH3T3 cells was run as a control for mobility of the non-PX containing Tks5 isoforms (asterisks). (B) Quantification of three independent experiments using densitometric analysis of the 130 kDa band. Average pixel intensity (arbitrary units) and standard deviations are shown. (*) p<0.001; (**) p<0.0005; (***) p<0.005 using Student's t test.

Since protein tyrosine phosphorylation may target proteins for degradation by the proteasome pathway, we treated Src-transformed cells expressing exogenous Tks5β with the proteasome inhibitor MG132. Src-transformed cells were very sensitive to the effects of this inhibitor and died within a few hours of treatment, making the results difficult to interpret. To overcome this issue, we overexpressed Tks5β in 293T cells and analyzed the effect of three different proteasome inhibitors (MG132, epoxomicin and bortezomib) on the expression of this Tks5 isoform. Accumulation of the overexpressed Tks5β was observed upon treatment with all three inhibitors (Figure 7 A, B). Taken together, these findings suggest that Tks5β protein stability is tightly regulated by the proteasome and that tyrosine phosphorylation by Src family kinases and/or other kinases might target Tks5β to degradation by the proteasome under conditions of elevated Src activity.

## Discussion

In this study, we describe two main findings: the essentiality of the PX domain-containing Tks5 isoform for proper mammalian development, and the existence of an additional isoform of Tks5, named Tks5β, which lacks the PX domain and is originated by alternative transcription.

First, we describe here that disruption of the mouse *sh3pxd2a* gene through the insertion of a gene-trap cassette between exons 1 and 2 led to the absence of the Tks5α/Tks5_long_ protein isoform, which was associated with complete cleft of the secondary palate and with neonatal death. The occurrence of this phenotype was affected by the genetic background, increasing from 50% in the C57BL/6JX129/SvJ background to 90% in the pure C57BL/6J background. We note that 30% of the Tks5α/Tks5_long_ pups produced on the mixed genetic background died in the neonatal period even though cleft palate was not evident; the reason for this would require further investigation. In humans, clefts of the lip and palate are the most frequent craniofacial defect affecting 1 in 700 births, and, although rarely lethal, they are associated with significant morbidity and financial cost derived from corrective surgeries and dental, speech and psychological treatments [7], [33], [34], [35]. Cleft palate can appear associated with other defects as part of a syndrome, usually of genetic origin and Mendelian inheritance, or as non-syndromic isolated trait in which both genetic and environmental factors contribute to create a complex etiology difficult to study [7], [8], [33], [36]. We identified the presence of cleft palate mainly as an isolated phenotype in the Tks5α/Tks5_long_ null mice, with the exception of sporadic bifid xiphoid cartilage of the sternum. However, we cannot predict if absence of TKS5α/TKS5_long_ could contribute to the etiology of syndromic or non-syndromic cleft palate in humans, since mutation of the *sh3pxd2a* gene is inherited in a Mendelian fashion as most syndromes are. On this basis, it could be possible that TKS5α/TKS5_long_ mutation is the origin of one of the 752 diseases of Mendelian inheritance orphan of a cause described at the OMIM. In mammals, the secondary palate formation takes place early in development (seventh week in humans and E11.5 in mice) when the palatal shelves emerge from the maxillary process [37]. The palatal shelves are cranial neural crest cell-derived mesenchymal protuberances covered by ectoderm-derived epithelium [38], [39], and alterations in growth, proliferation, movement, adhesion and death of cells composing the palatal structures can affect the palate architecture [37]. We have previously demonstrated that Tks5 and its phosphorylation by Src are necessary for neural crest cell migration *in vitro* and in zebrafish embryos, where disruption of Tks5 causes aberrant formation of the ceratobranchials, palatoquadrate, ethmoid plate and Meckel's cartilage, all cranial neural crest cell-derived structures [24]. Thus, it is possible that, in the mouse Tks5α/Tks5_long_ is involved in migration of cranial neural crest cells or their derivatives, affecting palatogenesis when absent. Given that Tks5 is essential for invadosome formation, including podosomes in neural crest cells [24], and that these cell structures are important in cell migration [12], we hypothesize that absence of Tks5α/Tks5_long_ would disrupt podosome formation during palatogenesis affecting cell migration and ultimately leading to cleft palate. Accordingly, absence of other invadosome proteins *in vivo* causes cleft palate. Loss of Filamin A, which is required for podosome stabilization during macrophage mesenchymal migration [40] and localizes to podosomes in osteoclasts [41] and to invadopodia in oral carcinoma cells [42], is associated with cleft palate in mice [43] and in humans [44]. Likewise, TGFβ2 knock-out mice present cleft palate as part of an array of defects affecting neural crest-derived structures [45]. TGFβ2 has been recently described to induce podosomes in human trabecular meshwork cells [46]. The present study also reveals a more general role for Tks adaptor function during mammalian development, because when both proteins are absent fetuses die *in utero* post-implantation, in sharp contrast with the Mendelian ratios achieved at birth for mice mutant for either Tks5α/Tks5_long_ or Tks4 [23]. Future work will be needed to discern if Tks4 and Tks5 have compensatory or redundant roles during development [27], and if podosomes are involved in Tks4 and Tks5 developmental functions in mammals.

The second main finding described here is the identification of a novel Tks5 isoform, named Tks5β, lacking the PX-domain. We identified Tks5β based on the following evidence: a) only the highest molecular weight band was missing from the characteristic Tks5 triplet in extracts from MEFs homozygous for the trapping mutation; and b) we identified two human ESTs in the GenBank database, DC332430 and BP219917, that have a 5′ end 134 bp and 119 bp, respectively, upstream of the beginning of exon 6, and both possess an in-frame ATG codon located 29 bp upstream of the beginning of that exon, indicating they encode proteins whose amino terminus starts in intron V, therefore lacking the PX-domain. Based on the high homology between the human *SH3PXD2A* and mouse *sh3pxd2a* genes, we designed a 5′RACE strategy that yielded a transcript in mouse NIH3T3 fibroblasts whose coding sequence started at the same ATG codon 29 bp upstream exon 6. We concluded this 29 bp sequence and exon 6 form a new 58 bp-long exon named exon 6β, encoding the amino terminus of this new isoform named Tks5β. The fact that we have been able to isolate a 5′RACE clone and specifically detect Tks5β mRNA in mouse tissues by RT-qPCR strongly points to the existence of a second alternative promoter located in intron V. In support of this, we have been able to identify a CpG island containing several SP1 transcription factor binding site consensus sequences within −400 bp from the beginning of exon 6β (data not shown). Also, we have identified 2 other GenBank ESTs sequences in human (FLJ56087) and gibbon (XM_003255430.1), with a 5′ end starting 131 bp upstream of the beginning of exon 6β. Curiously, the coding sequence of these 2 ESTs is located +5bp within exon 6 and continues to a 21 bp sequence in intron VI just upstream the beginning of exon 7, suggesting the existence of a new exon 65 bp-long containing exon 7 and the 21 bp upstream sequence. We have to add to these data the existence of Tks5_short_, another PX domain-lacking isoform identified in mouse that starts with a unique sequence located in intron VII and contains exons 8 to 15. 5′RACE and chromatin immunoprecipitation sequencing experiments suggest this form is transcribed from a different promoter than the one originating the Tks5α/Tks5_long_ isoform [31]. Together, all this evidence points to the existence of a second transcription initiation site hotspot in the *sh3pxd2a* gene, maintained across mammalian orthologues and located between intron V and exon 8, that would originate multiple isoforms each with a different short linear motif immediately preceding the first SH3 domain, and each lacking the PX domain. These might be driven by a second promoter that, based on our findings, would be located on intron V, although we cannot rule out the existence of other promoters in intron VI or VII [31]. The existence of alternative promoters in mammalian genes is a fairly common event, with an estimated frequency of 30 to 50% of human genes [47], [48], [49] and 50% of mouse genes [50]. Their presence is more frequent in genes involved in development [50] and in signal transduction [47], possibly allowing the generation of multiple tuned transcripts from single gene loci with diverse functional, temporal and/or spatial requirements. Importantly, abnormal use of alternative promoters has been associated with cancer [51]. In this regard, an altered balance between Tks5_short_ and Tks5α/Tks5_long_ expression has been linked to poor prognosis in human lung cancer [31], and it could be possible that Tks5β could also have a role in tumor suppression.

Alternative splicing can also contribute substantially to the diversity of Tks5 transcripts. Exons 7 and 10 are subject to alternative splicing [9]. Although the predominant forms for Tks5α/Tks5_long_ and Tks5β in mouse tissues and cells are the ones lacking exon 7 and containing exon 10, the existence of ESTs DC332430, FLJ56087 and XM_003255430.1, all with exon 7 but without exon 10, demonstrates that other alternative splicing isoforms exist. Together, these findings indicate the transcriptional complexity of the *sh3pxd2a* locus, which can potentially originate at least 12 different mRNA transcripts by alternative splicing and possibly by alternative promoter usage. If the existence of a second promoter is confirmed, the number of potential *sh3pxd2a* transcripts will be most likely reduced, since it has been shown that the activity of alternative promoters conditions alternative splicing [52], [53]. In any case, it remains to be elucidated if all the potential *sh3pxd2a* transcripts are found *in vivo*, and, in that case, if all of them are translated into Tks5 protein isoforms.

The short size of the unique distinctive sequence of Tks5β (29 bp/10 amino acids) impedes the generation of specific molecular tools, such as a riboprobe or an antibody, which would facilitate its study. However, the unique sequence is long enough to design primers for specific detection of its mRNA, whose expression we analyzed in adult mouse tissues and MEFs. We detected Tks5β in all tissues analyzed, although its relative expression to the Tks5α/Tks5_long_ expression is between 490 and 8 times lower. This indicates that the basal expression mRNAs of both isoforms is very different across tissues and suggests specific tissue-dependent regulation for each isoform. Interestingly, we also detected Tks5β mRNA expression in all tissues from Tks5α/Tks5_long_ null adult mice, while Tks5α/Tks5_long_ mRNA expression in these mice was absent or near undetectable, as expected. Notably, comparison of Tks5β mRNA expression between wild type and null mice tissues suggest a cross-regulation between Tks5α/Tks5_long_ and Tks5β transcript abundance. That cross-regulation seems tissue specific, since liver and spleen in wild-type mice have increased expression of Tks5β mRNA, while hearts from mutant mice have increased Tks5β mRNA expression with respect to wild-types, and the rest of tissues analyzed have a similar expression regardless of Tks5α/Tks5_long_ presence. While auto-regulation of gene expression is a common phenomenon conserved in biology [54], either by affecting transcription [55] or mRNA stability [56], [57], and there are examples describing how different protein isoforms originating from the same locus can modulate each other's biological functions [58], we cannot find examples of isoform cross-regulation at the transcriptional or mRNA processing levels. More experiments are needed to define the precise tissue and cellular expression of Tks5β in different physiological and pathological conditions, as well as the factors controlling its expression.

We cloned the mouse Tks5β form and verified that it corresponds to the 130 and 140 kDa bands we detect by immunoblotting of the endogenous Tks5 proteins. The over-expressed Tks5_short_ mostly corresponds to the 140 kDa band in Src-transformed cells. From our studies, however, we cannot assign a specific identity to each of the lower bands since cellular background and/or post-transcriptional modifications (e.g. phosphorylation or ubiquitination) may affect the mobility of the PX-lacking Tks5 forms. Furthermore, each band may correspond to more than one isoform and/or their post-translational modifications. Future analysis of the genomic regulation of *sh3pxd2a* gene will help elucidate the precise mechanism of Tks5 isoform expression. We could determine *in vitro* that Tks5β protein stability is regulated by Src activity. Src-transformation of NIH3T3 fibroblasts or MEFs causes an increased expression of the 150 kDa band of the Tks5 triplet (Tks5α/Tks5_long_) and a near disappearance of the 140 and 130 kDa bands, which was abrogated after treatment with Src-kinase family inhibitors. Tks5 was originally cloned from a library screen for new Src substrates [9], and the phosphorylation sites Tyr557 and Tyr619 targeted by Src activity are present in Tks5α/Tks5_long_ and Tks5β. Indeed, we observed that Tks5β isoform is actively tyrosine-phosphorylated in Src-transformed NIH3T3 cells, and that the degree of phosphorylation correlates with the stability of Tks5β, since treatment with phosphatase caused an increase in the amount of Tks5β. Moreover, we determined that Tks5β is targeted to degradation by the proteasome. MG132 was less efficient than epoxomicin and bortezomib in these assays, perhaps because MG132 decreased cell survival. Taken together, we hypothesize that Src tyrosine-phosphorylation of Tks5β actively targets this isoform for degradation by the proteasome. Interestingly, Src kinase activity can target a number of substrates for degradation by the ubiquitin-proteasome pathway [59], [60], [61], [62], [63], either by direct tyrosine phosphorylation of the proteins to degrade [60], [62], [63], or indirectly through the activation of the Ras-Raf-Mek1/2-Erk1/2 pathway [61]. Therefore, our results are compatible with the hypothesis that Src tyrosine phosphorylation of Tks5β controls its stability by making it a substrate for the proteasome pathway. In this manner Src would become a master regulator of the relative abundance of the Tks5α/Tks5_long_ and Tks5β isoforms at the post-translational level. In contrast, overexpressed Tks5_short_ protein does not seem to be affected by the presence of active Src. The biochemical bases for the difference in protein stability between the Tks5β and Tks5_short_ isoforms is currently unknown. In the future, it would be interesting to address whether the differential expression of the Tks5α/Tks5_long_ and Tks5β isoforms play a role in Src-driven physiological or pathological processes such as bone remodeling [64], embryonic stem cell differentiation [65] and cancer [66].

In summary, our findings demonstrate an essential role for Tks5α/Tks5_long_ in mouse development and the existence of Tks5β, a novel truncated isoform of the *sh3pxd2a* gene. The occurrence of Tks5α/Tks5_long_ and Tks5β, together with Tks5_short_ and several ESTs predicted to translate into PX domain-lacking Tks5 isoforms, indicates the transcriptional complexity of the *sh3pxd2a* gene, which appears conserved across mammalian species. Such an array of isoforms would only hint to multiple and fine-tuned functions of Tks5 in the cell. Further experiments will be needed to unveil the regulatory pathways controlling the expression of the *sh3pxd2a* gene and the cellular functions and biological implications of each of the Tks5 isoforms.
